# Influence of Environmental Factors on Phage–Bacteria Interaction and on the Efficacy and Infectivity of Phage P100

**DOI:** 10.3389/fmicb.2016.01152

**Published:** 2016-07-28

**Authors:** Susanne Fister, Christian Robben, Anna K. Witte, Dagmar Schoder, Martin Wagner, Peter Rossmanith

**Affiliations:** ^1^Christian Doppler Laboratory for Monitoring of Microbial Contaminants, Institute for Milk Hygiene, Milk Technology and Food Science, Department for Farm Animals and Public Veterinary Health, University of Veterinary MedicineVienna, Austria; ^2^Institute for Milk Hygiene, Milk Technology and Food Science, Department for Farm Animals and Public Veterinary Health, University of Veterinary MedicineVienna, Austria

**Keywords:** bacteriophage P100, host–phage interaction, environmental influence, resistance, *L. monocytogenes*, Listex^TM^ P100

## Abstract

When using bacteriophages to control food-borne bacteria in food production plants and processed food, it is crucial to consider that environmental conditions influence their stability. These conditions can also affect the physiological state of bacteria and consequently host–virus interaction and the effectiveness of the phage ability to reduce bacteria numbers. In this study we investigated the stability, binding, and replication capability of phage P100 and its efficacy to control *Listeria monocytogenes* under conditions typically encountered in dairy plants. The influences of SDS, Lutensol AO 7, salt, smear water, and different temperatures were investigated. Results indicate that phage P100 is stable and able to bind to the host under most conditions tested. Replication was dependent upon the growth of *L. monocytogenes* and efficacy was higher when bacterial growth was reduced by certain environmental conditions. In long-term experiments at different temperatures phages were initially able to reduce bacteria up to seven log_10_ units after 2 weeks at 4°C. However, thereafter, re-growth and development of phage-resistant *L. monocytogenes* isolates were encountered.

## Introduction

Although bacteriophages have been known for about 100 years, commercial use of lytic bacteriophages to detect and control pathogenic bacteria has increased in recent years (Mann, 2005). In particular, the use of phages as an alternate class of antibacterial agents against food-borne pathogens is of growing interest due to advantages that they offer. These include high host specificity and the fact that phages do not change the quality and sensory perceptions of food (Hagens and Loessner, 2007, 2010; Mahony et al., 2011).

Phages and phage products are now commercially available against nearly all important food-borne bacteria. A phage that is commonly used to combat *Listeria monocytogenes* is P100. This phage was originally isolated from the waste water of a dairy plant and is commercially available as Listex^TM^ P100. It has now been confirmed Generally Regarded As Safe (GRAS) by the US Food and Drug Administration (FDA; EFSA, 2009).

*Listeria monocytogenes* is one of the most important food-borne pathogens. Due to its ability to grow or persist at low pH values, high salt concentrations, low temperatures and in environments with low water activity, it is frequently found in adverse environments, such as food production plants (Koutsoumanis and Sofos, 2005; Swaminathan and Gerner-Smidt, 2007). When phages are used to combat bacteria in food production plants it is crucial to consider that factors such as heat, cold, dryness, nutrient deficit, curing, and exposure to chemical detergents or disinfectants influence phage stability, the physiological state of bacteria and consequently host–virus interactions (for instance attachment and replication of the phage) and the effectiveness of phages at reducing bacteria numbers (Ganegama Arachchi et al., 2013a,b; Chaturongakul and Ounjai, 2014; Denes and Wiedmann, 2014). In food production plants external factors such as temperature, pH, and water activity particularly affect the success of phage treatments (Garcia et al., 2008; Jończyk et al., 2011; Ly-Chatain, 2014).

The first requirement for the successful application of phages for biocontrol of bacteria is phage stability. Some authors assume a relationship between a phage’s morphology and its occurrence and infectivity in adverse environments, but evidence supportive of that hypothesis and predictions that can be made is lacking (Lasobras et al., 1997). In general, bacteriophage stability is highly variable and sensitivity of individual phage classes is highly diversified (Jończyk et al., 2011). It is known that the stability of a virus can be affected by changes of its virion or viral nucleic acid structure by various factors such as pH, ionic strength of the immediate environment, UV-light or heat (Maura and Debarbieux, 2011; Ly-Chatain, 2014). However, bacteriophages are the most abundant biological entities on earth, play a major role on nutrition, energy and global biogeochemical cycles (Fuhrman, 1999; Wommack and Colwell, 2000; Breitbart et al., 2002; Chibani-Chennoufi et al., 2004; Breitbart and Rohwer, 2005; Mann, 2005) and consequently are found in a range of adverse environments which theoretically could affect phage stability (Jończyk et al., 2011). For instance phages were found in environments with high UV irradiation and heat as the Sahara (Prigent et al., 2005) or hot springs (Breitbart et al., 2004) or in environments with various pH values and ionic strenghts like in food (Nel et al., 1987; Lu et al., 2003; Pringsulaka et al., 2011), cheese factories (Bruttin et al., 1997), humans (Bachrach et al., 2003), soil (Williamson et al., 2003), and sewage (Havelaar and Hogeboom, 1984). Moreover, maintenance of phage populations usually requires the presence of the bacterial host, which is also influenced by environmental factors (Maura and Debarbieux, 2011). As mentioned, bacterial fitness and physiological states can change the host–virus interaction.

The first step in host–virus interaction is attachment. On one hand attachment of the phages to bacteria and the susceptibility of the host can be reduced when bacterial fitness is compromised. A change in the physiological status of bacteria can lead to transcriptional responses that influence cell wall structures, which serve as receptors for phages (Denes and Wiedmann, 2014). Modified phage receptors could complicate or even prevent binding of the phage to the host. On the other hand, external factors, such as the presence of whey proteins, can result in non-specific binding or trapping of the phages thereby reducing host adsorption rates (Gill et al., 2006a). Another factor worth keeping in mind when bacteriophages are used to control food-borne bacteria, is accessibility to the target bacteria. Accessibility to the host can be limited when phages are applied in solid matrices or when the phage concentration and time of application are not optimal (Ly-Chatain, 2014).

Following attachment of phages to the host, the phage genome is injected into the host cell and replication of the phage particle can commence. However, inadequate nutrition, poor environments and a switch to the stationary growth phase lead to decreased productivity of phage infection and to small burst size as phage replication is dependent on host cell growth (Chibani-Chennoufi et al., 2004; Denes and Wiedmann, 2014).

Even though attachment and replication have major influences on the effectiveness of phage treatments, external factors can directly affect the outcome of phage infections. For instance, the presence of inhibitory compounds such as antibodies, whey proteins or bacteriocins can reduce the effectiveness of phage treatments and can even lead to resistance against the phages used (Maura and Debarbieux, 2011; Tessema et al., 2011; Abedon, 2012; Meaden and Koskella, 2013; Vongkamjan et al., 2013; Chaturongakul and Ounjai, 2014; Ly-Chatain, 2014). Suboptimal application of phages can also lead to adaptation of the bacteria to the phage, the development of phage resistance and consequently ineffective treatment (Hagens and Loessner, 2010).

Although it is known that several factors influence the efficacy of phage treatments against food-borne pathogens, relevant studies detailing interactions are limited and current research is still at an early stage (Garcia et al., 2008; Ganegama Arachchi et al., 2013b; Vongkamjan et al., 2013). Until now it has not been clear as to what extent environmental factors influence bacterial susceptibility to phage infection (Denes and Wiedmann, 2014). Moreover, past failures in phage therapy were mostly caused by limited knowledge of phage biology (O’Flaherty et al., 2009). Therefore, some investigators recommend testing of phage persistence in the absence of target bacteria (Chan et al., 2013). Efficacy testing of phage treatments has been suggested for each type of application and under different environmental conditions on a case-by-case basis (Skurnik et al., 2007; Garcia et al., 2008; Ganegama Arachchi et al., 2013a).

Therefore, the main focus of this study was to test the effectiveness of phage P100 against *L. monocytogenes* under conditions that can be found in food-processing plants. The influence of various chemical and physical factors on the infectivity and persistence of phage P100 was tested. These included the effect of different temperatures, pH values, salt and detergent concentrations, smear water and *Fraser* broth on the stability preinfection, the attachment on the host, and replication after injection of viral nucleic acid. Moreover, the effectiveness of phage P100 at eliminating *L. monocytogenes* under the above conditions was investigated. Long-term infection experiments were also conducted to examine the development of resistant *L. monocytogenes* isolates at different temperatures.

## Materials and Methods

### Bacterial Strain, Phage, and Growth Conditions

The phage P100 susceptible *Listeri*a strain used in this study was *L. monocytogenes* reference strain ATCC BAA-679 (EGDe). To establish standardized conditions, bacteria were grown in tryptone soya broth (TSB) with 0.6% (w/v) yeast extract (OxoidLdt., Hampshire) at 37°C. Overnight cultures were 10-fold diluted in fresh medium and incubated at 37°C for 3–4 h to obtain a maximum number of viable cells in the logarithmic growth phase (log phase). Phage P100 was supplied as the commercial preparation Listex^TM^ P100 (EBI Food Safety Wageningen, Netherlands). PFU (plaque forming units) determination resulted in a phage titre of approximately 6 × 10^10^ PFU/ml. The Listex^TM^ P100 preparation was diluted in SM buffer (5.8 g NaCl, 2.4 g Tris HCl, 1.0 g gelatine add. 1,000 ml, pH 7.0) to yield phages at other concentrations.

### Influence of Environmental Factors on the Infectivity of P100

To investigate the persistence and infectivity of P100 in the dairy environment over a longer time period, phages were added to different smear water samples (5 × 10^10^ PFU/ml final concentration) and stored at 4 and 10°C. Two types of smear water were obtained from an Austrian dairy plant. Each smear water type was then split into four samples and each sample was pre-treated as follows: the first sample was the original untreated smear water obtained from the dairy plant. The second sample was autoclaved smear water. The third sample was smear water inoculated with *L. monocytogenes* EGDe (5 × 10^8^ CFU). The fourth sample was smear water supernatant after centrifuging for 5 min at 8,000 × *g* in order to remove bacterial or eukaryotic (e.g., yeast) cells. Phage infectivity was determined every 10 days over a total of 117 days using the double agar overlay plaque assay (Kropinski et al., 2009) as previously described (Fister et al., 2016). As a non-treatment control, phage infectivity following incubation in SM buffer was monitored. Each experiment with each type of smear water was performed in duplicate.

To test the short-time influence of chemicals on P100 stability in terms of infectivity, phages were incubated in TSB containing either 2 M NaCl (Fisher Scientific, Leics, UK), the detergents Lutensol AO 7 (BASF, Ludwigshafen, Germany) and sodium dodecyl sulfate (SDS; SIGMA-ALDRICH, Steinheim, Germany; 5% each) and TSB adjusted to the pH values of 2, 3, 4, 5, 6, 7, 8, 9, 10, 11, 12 using 0.5 M HCl or NaOH (Merck, Darmstadt, Germany). After 1, 6, and 24 h the number of phages was determined using the double agar overlay plaque assay. For comparison and as a non-treatment control, phages were incubated in TSB. Each experiment was carried out at least in triplicate on different days.

### Adsorption Tests

To determine binding and replication characteristics of P100, adsorption tests were performed under different environmental conditions. The variables were salt concentration, pH, concentration of detergents and temperature.

Log phase bacteria cultures were adjusted to an OD_600_ of 0.6. Thereafter bacteria were centrifuged at 8,000 × *g* for 5 min. The pellet was resuspended in TSB medium representing the chemical conditions described above (see Influence of Environmental Factors on the Infectivity of P100; pH 2 and 12 were not further tested as phage infectivity was reduced more than seven log_10_ units within 1 h). Phage P100 was added to a MOI (multiplicity of infection, ratio of phages to bacteria) of 0.1. In order to determine adsorption of the phages on the host under these conditions, the infection was stopped by the addition of ice-cold TSB followed by centrifugation for 2 min at 8,000 rpm after 0, 10, 20, 30, 40, 50, and 60 min as described by Wendlinger et al. (1996). Afterward, phages remaining in the supernatant were serially diluted in SM buffer and quantified using the double agar overlay plaque assay. To monitor the replication ability of the phage at the conditions described, the number of phages was measured after stopping the infection after 0, 1, 3, and 6 h (and after 12 and 24 h at pH 4). All experiments were carried out at least twice in duplicate.

### Chemical Influence on the Efficacy of Phage Treatment (Short-Term)

To analyze the influence of different chemical conditions on the efficacy of phage treatments, *L. monocytogenes* EGDe was infected with phage P100 and grown in *Fraser Listeria Selective Enrichment Broth* base (Merck, Darmstadt, Germany), TSB and TSB medium with different salt concentrations, detergent (Lutensol AO 7 and SDS) concentrations and in TSB adjusted to different pH values (see Influence of Environmental Factors on the Infectivity of P100). Additionally, the efficacy of phage treatment in smear water, obtained from an Austrian dairy plant, was investigated. The influence of these factors on the growth of uninfected *L. monocytogenes* EGDe was monitored as a control.

In detail, two concentrations of log-phase *L. monocytogenes* EGDe cultures (approximately 2.5 × 10^7^ and 2.5 × 10^6^ CFU/ml, 1:10 and 1:100 dilutions of a OD_610_ = 0.6 log phase culture, respectively) were infected with P100, with a MOI of 10, and were incubated at 37°C in TSB media representing the chemical conditions described above. The OD_610_ of these infections was measured for 24 h every hour in a TECAN F100 microplate reader (Tecan Austria GmbH., Groeding, Austria). The experiments were performed at least twice on different days and in duplicate.

### Influence of Temperature on the Efficacy of P100 Treatments (Long-Term)

To examine the influence of temperature on the efficacy of phage treatments, 500 μl of a log phase *L. monocytogenes* EGDe culture was incubated with an equal volume of Listex^TM^ P100 dilutions (2 × 10^10^ and 2 × 10^9^ PFU/ml, resulting in MOIs of 10 and 100) for 30 min at room temperature. Thereafter, 5 ml of TSB was added and the infected *Listeria* stored at 4, 10, and 20°C for 17 weeks. The number of surviving bacteria was determined weekly using the plate count method and compared to a non-infected control of the same isolate (treated with SM buffer instead of Listex^TM^ P100). All plating was performed in duplicate on TSA with at least two different dilutions and plates were incubated overnight at 37°C. The experiment was performed twice.

### Screening for Newly Formed Resistances and Confirmation of Insensitive Isolates

When re-growth of *L. monocytogenes* in the long-term temperature experiments (Influence of Temperature on the Efficacy of P100 Treatments (Long-Term)) was observed, single colonies growing on the TSA plates of the experiment were selected and used for preparation of overnight cultures. These cultures were then used to screen for insensitive *L. monocytogenes* isolates using cross streak tests (Miller, 1998) as follows: 50 μl P100 (6 × 10^10^ PFU/ml) were allowed to run from the top to the bottom of TSA+Y plates. After drying, the streak was crossed with the cultures of re-growing *L. monocytogenes* isolates. *L. monocytogenes* EGDe was used as sensitive control. Plates were incubated overnight at 37°C. Suspicious isolates, which grew in the phage-zone of the plates, were confirmed as *L. monocytogenes* by plating on selective ALOA and PALCAM agar. Additionally, suspicious isolates were confirmed as *L. monocytogenes* by PCR as described by Rossmanith et al. (2006). Resistance and reduced susceptibility were confirmed by small drop plaque assays (Mazzocco et al., 2009). Stability of resistance was tested by passaging the isolates for five passages.

## Results

### Phage P100 Is Stable under Most Tested Chemical Conditions

**FIGURE 1:**
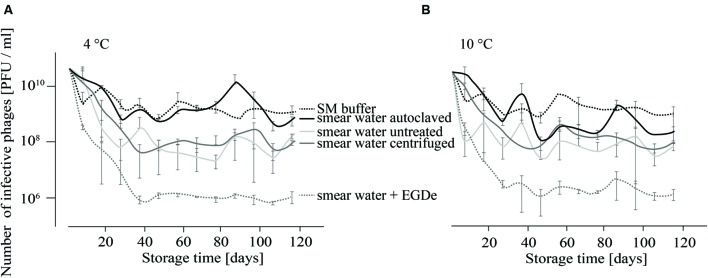
**Infectivity of P100 stored in smear water and SM buffer at 4°C (A) and 10°C (B) for 117 days.** Smear water was either untreated (as obtained from the dairy plant), autoclaved or centrifuged in order to remove bacterial or eukaryotic (e.g., yeast) cells. Further, *L. monocytogenes* EGDe was added to one smear water sample. Experiments were done twice in duplicates and mean values and standard deviations were shown.

For the investigation of P100 stability in SM buffer and different types of smear water over a period of 4 months, comparison revealed similar results for samples that were stored at 4 and 10°C (**Figures 1A,B**). At both incubation temperatures the decrease in the number of infective phages was lowest when P100 was stored in SM buffer, followed by phages stored in autoclaved smear water. In both experimental conditions, the number of phages decreased within the first month by about 1–2 log_10_ units and did not change distinctly until the end of the investigation. Phages stored in untreated smear water were reduced by about 2.5–3 log_10_ units. The highest phage reduction was observed in smear water containing *L. monocytogenes* EGDe. In this case P100 was reduced by four log_10_ units.

As a next step in the short-term experiments, phage P100 was incubated in TSB containing different detergents or adjusted to different pH values since food can have a wide range of pH values. Infectivity of P100 was then determined after 1, 6, and 24 h of incubation (**Table 1**). At pH 2, P100 numbers were rapidly reduced below the detection limit, indicating a reduction of at least 7–8 log_10_ units within 1 h. At pH 3, P100 concentrations decreased within 1 h by at least 5.4 log_10_ units and P100 numbers were reduced below the detection limit (7–8 log_10_) at 6 h. At pH 4–10, P100 numbers were not distinctly reduced and even after 24 h the decrease in phage number was ≤0.5 log_10_ units. At pH 11 phage numbers were reduced by nearly 1 log_10_ level within 1 h. After 24 h, infectivity of phages was also reduced by about 1 log_10_ unit compared to the control. Five percent Lutensol AO 7 did not change the phage number within 1 day. The second tested detergent, SDS, reduced P100 numbers by about 0.3 log_10_ units, when the SDS concentration in TSB was 5%. After 6 and 24 h of incubation, the phage concentration was decreased by 0.6 and 1.2 log_10_ units.

**Table 1:** Reduction of infectivity of P100 after incubation in TSB adjusted to different pH values, containing NaCl or detergents.

Chemical condition		Incubation time	
	
	1 h	6 h	24 h
pH 2	≥7	≥7	≥7
pH 3	5.4 -≥ 7	≥7	≥7
pH 4	0.1 (±0.1)^a^	0.4 (±0.5)	0.1 (±0.2)
pH 5–10	≤0.2	≤0.3	≤0.5
pH 11	0.8 (±1.4)	0.1 (±0.1)	0.9 (±1.1)
pH 12	≥7	≥7	≥7
2 M NaCl	≤0.5	≤0.5	≤0.5
5% Lutensol AO 7	0.0 (±0.1)	-0.1 (±0.8)	-0.1 (±0.2)
5% SDS	0.3 (±0.3)	0.6 (±0.2)	1.2 (±0.7)

*^a^log_10_ units reduction (± standard deviation).*

*≥7: higher than the detection limit (indicating a reduction of ≥7 log_10_ units).*

### Binding of Phage P100 Was Possible, but Its Replication Was Not Observed at all Tested Chemical Conditions

**FIGURE 2:**
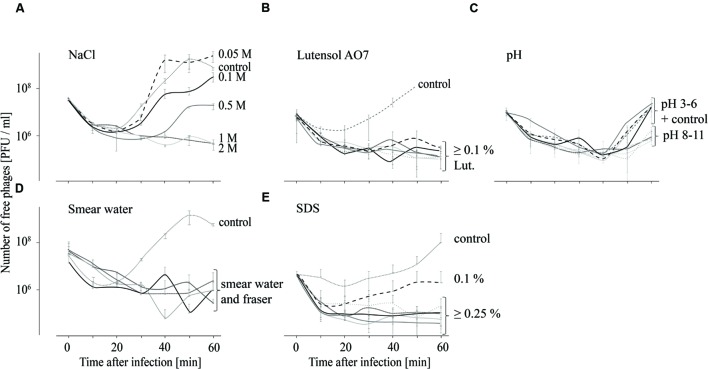
**Adsorption tests performed over 60 min indicating attachment (decreasing number of free phages) of phage P100 to *L. monocytogenes* in TSB containing NaCl (A), Lutensol AO 7 (B), TSB adjusted to pH values 3–11 (C), smear water and *Fraser* (D), and TSB containing SDS (E).** All experiments were done twice and in duplicates and mean values and standard deviations were shown.

In order to test the influence of different chemical conditions on the attachment of P100 to *L. monocytogenes* EGDe, adsorption tests in different smear water samples, in *Fraser* medium, in TSB media containing different NaCl or detergent concentrations and in TSB media with different pH values were performed and the respective results are shown in **Figure 2**. For all tested incubation conditions distinct binding of phage P100 to *L. monocytogenes*, indicated by an initial drop in free phage counts in the supernatant, was found. At NaCl concentrations up to 0.1 M an increase of free phages in the supernatant was observed after 30 min (**Figure 2A**), indicating replication of P100. At 0.5 M NaCl the number of unattached phages increased after 40 min and at 1 and 2 M NaCl no increase in free phage numbers was observed within 60 min. In Lutensol and SDS-containing TSB medium, in all tested smear water samples and in the selective *Fraser* medium, there was also no detected increase in unattached phages within 1 h, except in the presence of 0.1% SDS (**Figures 2B,D,E**). At all tested pH values an increase of free phages was observed 40 min after infection (**Figure 2C**). The number of unattached phages 60 min after infection was lowest at pH values 3 and between 8 and 11.

**FIGURE 3:**
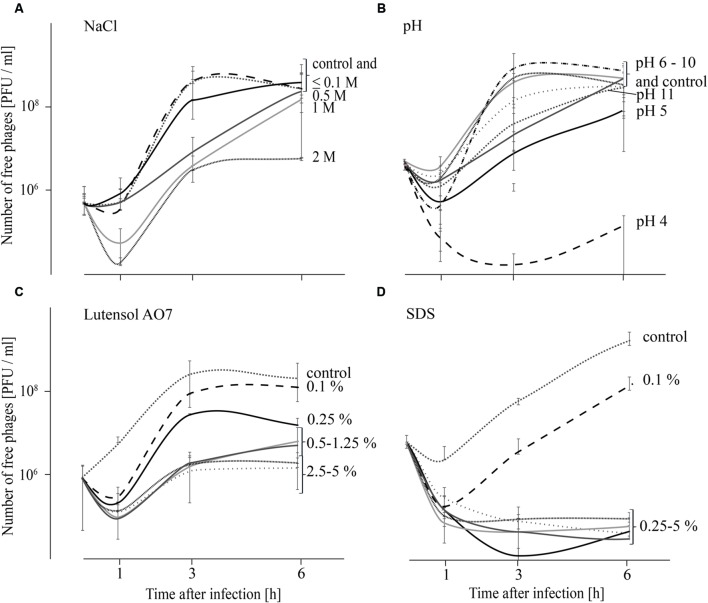
**Adsorption tests performed over 6 h in TSB media containing NaCl (A), adjusted to different pH values (B) and containing the detergents Lutensol AO 7 (C) or SDS (D).** Graphs show the number of free (unattached extracellular) phages. All experiments were carried out twice and in duplicates and mean values and standard deviations are shown.

As replication of phages was not observed at all tested conditions within 1 h after infection, adsorption tests were also performed over 6 h in order to obtain an indication if phage replication at different chemical conditions (described above) is possible. Results indicate that replication of phages is not possible at all tested conditions (**Figure 3**) although phage stability was not strongly impaired at all tested conditions within the first 6 h (**Table 1**). When the influence of salt was tested, the number of free phages was very similar in TSB media containing up to 0.1 M NaCl compared with the TSB control (**Figure 3A**). The number of free phages in TSB containing 0.5 or 1 M NaCl distinctly increased later compared with the TSB control. In TSB media containing 2 M NaCl an increase in free phages was observed after 1 h. However, after 6 h phage concentration was similar to that at the beginning of the experiment. These results indicate that replication of P100 is influenced by increasing concentrations of NaCl. Adsorption tests in TSB media with pH values ranging from 5 to 11 indicate that replication of phage P100 in *L. monocytogenes* is possible (**Figure 3B**). At pH value 4 the number of free phages decreased for up to 3 h after infection. Thereafter, there was a slight increase in phages up to 6 h after infection. The concentration of phages measured at the beginning of the experiment was not reached indicating no replication of phage P100 at pH 4. Even 12 or 24 h after infection the number of free phages at pH 4 did not increase indicating that no replication was possible (Supplementary Figure S1). The effect of detergents on replication of phage P100 was tested by performing adsorption tests in TSB media containing 0.1–5% Lutensol AO 7 or SDS (**Figures 3C,D**). At all Lutensol AO 7 concentrations (≤0.25%) the number of free phages increased between 1 and 3 h, indicating that P100 replication is possible. In TSB media containing 0.5–5% Lutensol AO 7, the concentration of free phages also increased between 1 and 3 h, but after 6 h the phage concentrations had still not reached initial values. In the presence of the second detergent, SDS, no increase of free phages was observed when concentrations ≥0.25% are present (**Figure 3D**). These data indicate that phage replication did not take place. In contrast, in TSB containing 0.1% SDS phage numbers were observed to increase after 1 h, indicating replication of P100.

### Chemical Conditions that Reduce the Growth of *L. monocytogenes* Lead to Higher Efficacy of Phage Treatments

**FIGURE 4:**
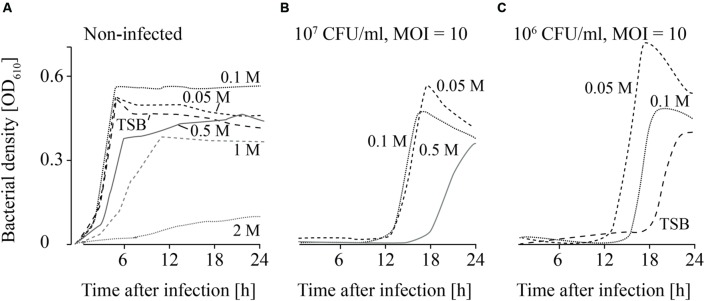
**Growth curves of uninfected (A) and infected (B,C; MOI = 10) *L. monocytogenes* in TSB and NaCl containing TSB media.** Bacteria concentrations at the beginning of the infections were 10^7^ CFU/ml **(A,B)** and 10^6^ CFU/ml **(C)**. All NaCl concentrations (0–2 M) were tested. For the sake of clarity only the curves of growing bacteria were demonstrated while the others on base line level were not depicted. Moreover, one of four independent experiments is representatively shown.

**FIGURE 5:**
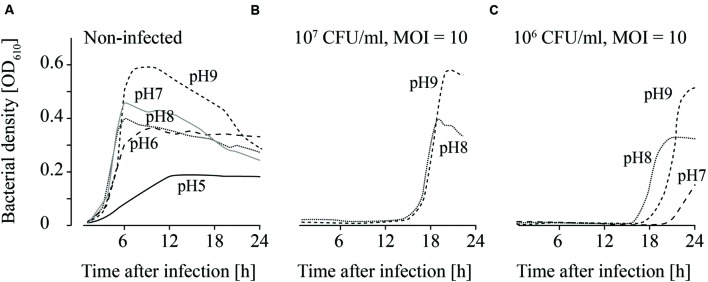
**Growth of uninfected (A) and infected (B,C; MOI = 10) *L. monocytogenes* in TSB adjusted to different pH values.** The bacteria concentrations at the beginning of the infections were 10^7^ CFU/ml **(A)** and **(B)** and 10^6^ CFU/ml **(C)**. All pH values (4–11) were tested. For the sake of clarity only the curves of growing bacteria were demonstrated while the others on base line level were not depicted. Moreover, one of four independent experiments is representatively shown.

**FIGURE 6:**
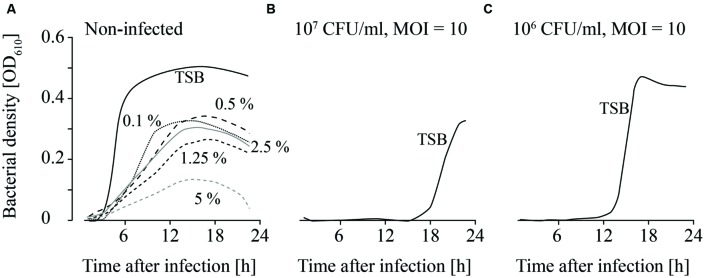
**Growth of uninfected (A) and infected (B,C; MOI = 10) *L. monocytogenes* in TSB containing different Lutensol AO 7 concentrations.** Bacteria concentrations at the beginning of the infections were 10^7^ CFU/ml **(A,B)** and 10^6^ CFU/ml **(C)**. All concentrations (0–5%) were tested. For the sake of clarity only the curves of growing bacteria were demonstrated while the others on base line level were not depicted. Moreover, one of four independent experiments is representatively shown.

In order to test the effect of different chemical conditions on the efficacy of P100 to reduce *L. monocytogenes* EGDe, bacteria (2.5 × 10^7^ and 2.5 × 10^6^ CFU/ml) were infected with P100 with a MOI of 10 and the growth of bacteria in TSB media adjusted to different pH values or containing different NaCl or detergent concentrations was monitored by measurement of OD_610_ over 24 h. All experiments were carried out twice and in duplicate. In some cases, growth of *L. monocytogenes* was observed at shifted time points or there was growth in one experiment, but not when repeated. In Supplementary Figures S2–S4 each single growth curve that was measured is shown. For the sake of simplicity **Figures 4–6** show representative growth curves for one measurement of each tested condition.

When the effects of *Fraser* medium (Supplementary Figures S2A–D) and NaCl were tested on the growth of uninfected *L. monocytogenes*, a distinct reduction in growth was only observed in TSB medium containing 2 M NaCl (**Figure 4A**; Supplementary Figure S2). When 10^7^ CFU/ml were infected with phage P100 (MOI = 10), growth of *L. monocytogenes* was suppressed in TSB and TSB containing 1 and 2 M NaCl (**Figure 4B**). Growth of *L. monocytogenes* in TSB containing between 0.05 and 0.5 M NaCl and in *Fraser* broth (Supplementary Figures S2E–H) was observed in all experiments. When lower bacteria concentrations (10^6^ CFU/ml) were infected, in three out of four experiments growth of *L. monocytogenes* was observed in *Fraser* medium (Supplementary Figures S2I–L), in 0.05 and 0.1 M NaCl containing TSB medium and in half of the experiments in TSB media (**Figure 4C**).

Uninfected *L. monocytogenes* were able to grow in TSB adjusted to pH values ranging from 6 to 9 (**Figure 5A**). Bacterial growth was also observed at pH 5 in three of four experiments, although lower OD values were obtained. When higher *L. monocytogenes* concentrations (10^7^ CFU/ml) were infected, phage P100 was not able to suppress growth of *L. monocytogenes* at pH values 8 and 9 longer than 12–20 h (**Figure 5B**). In one of four repetitions at pH 7, growth of *L. monocytogenes* was observed. When lower bacteria concentrations were infected (10^6^ CFU/ml), growth of *L. monocytogenes* was possible at pH 8 and 9 (**Figure 5C**). At pH 7 bacteria growth was monitored in two of four repetitions and at pH 6 in three of four repetitions.

Detergents showed the strongest impact on the growth of both, infected and non-infected, *L. monocytogenes*. *L. monocytogenes* was able to grow at all Lutensol AO 7 concentrations (0.1–5%) tested (**Figure 6A**). However, even at low Lutensol concentrations, growth of *L. monocytogenes* was distinctly reduced compared with growth in TSB media without Lutensol. When phages were added, growth was suppressed for at least 24 h at all concentrations tested (**Figures 6B,C**). The only exception was 0.1% Lutensol in TSB. Here, *L. monocytogenes* started to grow 15 h post-infection in one out of four replicates (Supplementary Figures S4I–L).

*L. monocytogenes* was not able to grow in TSB containing 0.1–5% SDS for the first 24 h (data not shown). Consequently, no infection experiments were performed.

### Development of Phage Insensitive *L. monocytogenes* Isolates Was Observed at all Tested Temperatures

As growth of *L. monocytogenes* at low temperatures is slow, the influence of temperature and host–phage ratio on the efficacy of P100 treatments was tested in longer-term experiments. *L. monocytogenes* EGDe was infected with MOIs of 10 and 100, incubated at 4, 10, and 20°C and the number of surviving bacteria determined weekly for 17 weeks.

**FIGURE 7:**
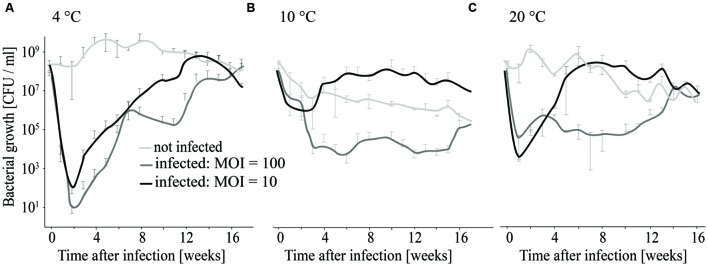
**Long-term infection experiment using different MOIs and temperatures for incubation.** Growth of infected and non-infected *L. monocytogenes* was monitored at 4°C **(A)**, 10°C **(B)**, and 20°C **(C)**.

At 4°C *L. monocytogenes* was reduced by six and seven log_10_ units (MOI = 10 and MOI = 100) within the first 2 weeks (**Figure 7A**). Thereafter re-growth was observed. At 10 and 20°C the reduction in *L. monocytogenes* counts was maximal at 4–5 log_10_ units and re-growth of *L. monocytogenes* was also observed (**Figures 7B,C**).

As described above, following an initial reduction of bacteria at the beginning of infection, re-growth of *L. monocytogenes* was observed at all tested temperatures and at both tested phage concentrations. In order to determine if survival and re-growing *L. monocytogenes* results from decreased efficacy of phage P100 or is caused by the resistance development, *L. monocytogenes* isolates were randomly selected at the end of the experiment. Afterward, they were tested for their susceptibility to P100. *L. monocytogenes* isolates that were obtained from uninfected samples were all susceptible to phage P100. In contrast, all *L. monocytogenes* isolates that were obtained from infected samples (independent of which MOI was used for infection and at which temperature the infected bacteria were incubated), had reduced susceptibility to P100 (at least four log_10_ units reduction compared to non-infected *L. monocytogene*s EGDe, see Supplementary Table S1). Moreover, susceptibility to the phage did not change over five passages, indicating that insensitivity to P100 was stable.

## Discussion

In food production bacteriophages have become more and more attractive as tools to eliminate *L. monocytogenes* (Guenther et al., 2009; Soni et al., 2010; Guenther and Loessner, 2011; Soni et al., 2012; Oliveira et al., 2014). In a recent publication Fister et al. (2016) investigated the basic issues regarding the occurrence of P100 resistant *L. monocytogenes* associated with the application of Listex^TM^. A connection between use of Listex^TM^ and the presence of resistant *L. monocytogenes* strains was demonstrated. Additionally, the host relationship was investigated *in vitro*.

However, *in vivo* especially in food production plants there are harsh environmental conditions. Therefore, this study took into account the influence of several of these chemical and physical factors on P100 that are typically encountered in the food-production environment. These conditions potentially influence the ability of P100 to reproduce by acting on the phage’s infection cycle. Relevant factors are: (i) the stability of the phage before infection, (ii) the attachment process after contact with the host, (iii) replication after attachment, injection of the phage genome, and finally (iv) the efficacy of the infection, according to reduction of bacterial cell numbers. These four parameters were used to analyze the efficacy of P100 against *L. monocytogenes*.

In following the sequence of events during the reproductive infection cycle of P100 we examined the influence of pH, salt concentration and the presence of two detergents as representative of chemical factors present in the production environment. The respective experiments were conducted short-term (up to 24 h). Moreover, long-term experiments were conducted for 120 days.

First of all the stability of the persisting phage particles was investigated, as loss of phage infectivity would result in a rapid decrease of overall efficacy of phage treatments (Gill et al., 2010). We tested the stability of phage P100 because during the cheese ripening process the pH increases and this consequently supports the growth of *Listeria* (Guenther and Loessner, 2011). In this study we did not observe a significant reduction in phage P100 numbers in TSB media over the pH range 4–10 within 24 h (**Table 1**).

These data indicate that P100 is stable over a wide pH range. In contrast, other phages were decreased on sliced apples due to the low pH (4.4) and were consequently not able to prevent microbial growth (Leverentz et al., 2003). Our results showed that P100 numbers rapidly decreased by >5 log_10_ units within 1 h, only when the pH values were ≤2 and ≥12. These results reflect the importance to test the stability of each phage individually and indicate that phage P100 can probably be inactivated by the use of disinfectants with very low or very high pH values.

As salt is one of the most frequent additions to food, and since it plays an important role in cheese production (brine wash contains 15–20% NaCl; Carlton et al., 2005), we tested the effect of various NaCl concentrations on phage P100. Salt is known to influence osmotic pressure, leading to breaks in phage heads and tails (Jończyk et al., 2011). However, in our study no reduction in phage infectivity was observed when P100 was stored for up to 24 h in TSB containing 2 M NaCl.

Cleaning and disinfection are also major ongoing activities in the food safety environment and in food production. The most commonly used detergents for cleaning are SDS and a group of detergents summarized as *Lutensols*. Lutensols are polymeric ethoxylated aliphatic alcohols that act as non-ionic tenside detergents, which means that they, like SDS, are surfactants. The particular advantage of Lutensols is that they are considered to be biodegradable and significantly less toxic than SDS (Smułek et al., 2015). In our study we found no reduction in phage P100 infectivity in the presence of up to 5% Lutensol AO 7 in TSB within 24 h, whereas 5% SDS reduced phage infectivity by about 1.2 log_10_ units within 24 h. This finding is in agreement with another study, in which it was speculated that SDS may be capable of denaturing the capsid proteins of non-enveloped viruses (Howett et al., 1999).

Smear water is a major application area for Listex^TM^. Therefore we tested the influence of smear water on the stability of P100 at different temperatures. As recommended by other authors, phage persistence with and without target bacteria was examined (Chan et al., 2013).

The infectivity of P100 was reduced faster in smear water than in SM buffer (**Figure 1**). The presence of *L. monocytogenes* increased this effect distinctly, which was expressed as 2 log_10_ units reduction in the PFU number. This could be due to different reasons. One reason could be that phages bind to the host cells and can therefore not be detected. Additionally, bacteria have several phage defense mechanisms which could reduce the P100 numbers. Moreover, microbial load in smear water may also provide non-specific binding sites (Garcia et al., 2008). In food stuffs or raw milk, phages can be entrapped by charge or hydrophobic interactions and it is known that some phages are inactivated by raw milk and bovine whey proteins (Gerba, 1984; Gill et al., 2006b, 2010). Non-specific binding and consequently phage trapping could also occur in smear water. Furthermore, proteolytic activity of the smear water could affect the infectivity and integrity of phage particles (Guenther and Loessner, 2011). Another reason might be bacteriocins, which are known to be produced by lactic acid bacteria that could be found in smear water during dairy product processing. Bacteriocin sakacin P may cause phenotypic and transcriptional changes in *L. monocytogenes* (Tessema et al., 2011), which could consequently effect the host–phage relationship. Interestingly, temperature differences did not distinctly change phage survival, although it is known that phages are generally more stable at lower temperatures (Adams, 1949; Gill et al., 2010).

Overall the data suggest that phage P100 is very stable under a variety of environmental conditions. Therefore, P100 is most likely able to remain infective for a long period of time in food production plants. Indeed, from another study we were able to detect phage P100 in a dairy plant 3 months after the cessation of Listex^TM^ P100 use (Rossmanith et al., 2014).

Besides phage stability, attachment of the phage to its target is the next crucial step in the phage infection process. Chemical and physiological factors on the other hand can influence the efficacy of phage binding. It is known that bovine whey proteins, which are also likely to be present in cheese and smear water, affect host adsorption of phages (Denes and Wiedmann, 2014). However, our data indicate that binding of P100 to *L. monocytogenes* is not affected by smear water (**Figure 2**). In addition, high NaCl concentrations (up to 2 M), which are commonly encountered in brine baths, do not hinder P100 binding to its host. Moreover, our data indicate that P100 binding is not influenced at all the pH values tested and in the presence of detergents such as SDS or Lutensol AO 7.

Phage replication is known to depend mainly on the physiological state of the host and the burst size correlates with the growth rate of the bacteria (Chibani-Chennoufi et al., 2004; Denes and Wiedmann, 2014). However, the growth rate is dependent upon stress conditions, which will frequently be present in food processing environments and thus pathogens are normally not found undergoing exponential growth (Denes and Wiedmann, 2014). However, metabolically active bacteria are a requirement for successful use of bacteriophages for active control strategies. These active attempts rely on host cell metabolism, which is necessary for phage replication and consequently active disruption of the bacterial cell leading to release of progeny phages. Results of our study have shown that replication was highly impaired (delayed or even inhibited) by chemical parameters such as pH value, high salt concentrations and the presence of surfactants, especially SDS (**Figure 3**). These parameters mainly influence the metabolic condition and growth rate of host cells, therefore hindering active control strategies (Denes and Wiedmann, 2014).

Although replication is necessary in the active approach for successful phage treatments, passive approaches in contrast do not depend on replication (Guenther et al., 2009; Hudson et al., 2010). In this case efficacy as defined by reduced bacterial cell counts is a relevant criterion for assessment and the fourth factor that was investigated in this study. Passive strategies are based on high phage concentrations (MOI ≥ 10; Kasman et al., 2002). On one hand phage infection should then lead to a change in host synthetic machinery resulting in arrest of host replication and a bacteriostatic effect. On the other hand a scenario termed “lysis from without” has been described (Abedon, 2011). This refers to infection of one bacteria cell with a high number of phages leading to its passive lysis. The supplier of Listex^TM^ P100 recommends an application of 1 × 10^8^ PFU/ml or 1 ml per 100 cm^2^ (= 2 × 10^11^ PFU/100 cm^2^; http://www.listex.eu/wp-content/uploads/Listex-Application-Data-Sheet-Cheese.pdf, accessed on 2016-05-16 and www.listex.eu/cheese-and-Listeria, accessed on 2015-03-07). Because in general the contamination rate of *L. monocytogenes* in food production pants is relatively low (maximum 10^7^ CFU/ml *L. monocytogenes* were reported in outbreak cases; (Farber and Losos, 1988)), the recommended phage concentrations are likely to be consistent with a passive treatment strategy. Therefore, we examined the effect of chemicals on the efficacy of phage treatments using infective doses of MOIs of at least 10. Results (**Figures 4–6**; Supplementary Material) indicated that bacterial growth is inhibited and delayed by the environmental conditions and enhanced the efficacy of phage treatment. This observation is in agreement with other authors who were able to show that phage treatments are more efficacious in combination with conventional disinfection and sanitation measures (Montanez-Izquierdo et al., 2012). Synergetic effects were, for instance, reported in combination with the bacteriocin nisin on melons and apples (Leverentz et al., 2003). However, they were not obtained on beef (Dykes and Moorhead, 2002). Furthermore, in this study the efficacy of phage P100 in *Fraser* broth was similar as in TSB. *Fraser* broth is frequently used for selective enrichment of *L. monocytogenes*. These results indicate that the presence of phages (which were applied in the production plant) can influence growth dependent standard monitoring methods and might even cause false negative results.

In our study, growth was monitored over 24 h. As proliferation of *Listeria* is generally slow at 4°C, long-term experiments (120 days) were also conducted to consider the effect of temperature. Results (**Figure 7**) of these experiments indicate that the highest reduction in *L. monocytogenes* counts was achieved at 4°C. However, at all temperatures tested, re-growth was observed. This is an interesting observation in respect of resistance development, which was confirmed by testing single colonies. At all tested temperatures, *L. monocytogenes* isolates were found that had a reduced sensitivity to phage P100 (**Figure 7**; Supplementary Table S1). This finding is in accordance with Guenther and Loessner (2011) who detected phage-insensitive clones when phage A511 was used for 22 days for the treatment of red mold cheese during ripening. In contrast, no phage insensitive clones were detected when they used another *L. monocytogenes* strain. In our study we used *L. monocytogenes* EGDe, because it is a frequently used model strain for *L. monocytogenes* serotype 1/2a, one of the most important food-borne serotype. Nevertheless, it is possible that some of our data are not directly applicable to all *L. monocytogenes* strains. However, in food production plants or natural environments there is always heterogeneity of bacteria and stress-resistant variants can be found (Abee et al., 2016). For example, in general *L. monocytogenes* serotypes 4 and 1/2 were more susceptible to phages compared to strains in other serotypes, however is was reported that in food production plants within one serotype both, phage susceptible and phage insensitive isolates, can be found (Vongkamjan et al., 2012, 2013; Fister et al., 2016). The development of phage resistant variants can be caused by different reasons. It is known that a single point mutation can result in changes of the P100 attachment site, leading to phage resistant *L. monocytogenes* (Prof. Loessner, personal communication). Moreover, alterations in the physiological status of bacteria can lead to transcriptional responses that ultimately lead to cell wall structure changes that reduce the susceptibility of the cell to the phage. Additionally, the permanent presence of phages in the production plant is likely to support development of phage-resistant bacteria (Hagens and Loessner, 2010; Hudson et al., 2010).

In summary, our data show that phage P100 is stable under most conditions typically encountered in dairy-production environments. Results also demonstrate that phage attachment is possible under all tested conditions. In contrast, phage replication, which is necessary for all active control strategies, is dependent upon host cell growth, which is reduced by factors such as the presence of detergents, extreme pH values or high salt concentrations. Our results demonstrate that high numbers of phages in combination with environmental conditions that limit growth of bacteria are most effective. The use of bacteriophages for biocontrol in food production plants has to be critically considered case by case. On one hand the efficacy of phage treatments could be reduced by the development of phage resistances. Moreover, growth-dependent microbiological standard monitoring methods (like growth in *Fraser* broth) can be influenced by the presence of phages.

## Author Contributions

Conception and design of the work: SF, DS, MW, PR; Acquisition of data: SF, CR; Analysis and Interpretation of data: SF, AKW, PR; Drafting the work: CR, SF, PR; Revision of the manuscript: SF, AKW, DS, MW, PR; All authors approved the version to be published in *Frontiers in Microbiology* and agreed to be accountable for all aspects of the work.

## Conflict of Interest Statement

The authors declare that the research was conducted in the absence of any commercial or financial relationships that could be construed as a potential conflict of interest.
