# Supplementary material for: Influence of Environmental Factors on Phage–Bacteria Interaction and on the Efficacy and Infectivity of Phage P100
Source: Front Microbiol. 2016 Jul 28;7:1152. doi: 10.3389/fmicb.2016.01152 (PMC4964841; doi:10.3389/fmicb.2016.01152)
Supplement: Supplementary file 1 [file Table_1.DOCX]

| Table S1: Isolates selected from long-term infection experiments | | | |  |
| --- | --- | --- | --- | --- |
|  |  |  |  |  |
| Isolate ID | Temperature | MOI | EOP^a^ |  |
|  |  |  |  |  |
|  |  |  |  |  |
| 4-C-I | 4 °C | not infected | 7.15E-01 |  |
| 4-100-I | 4 °C | 100 | <1.0E-8 |  |
| 4-100-II | 4 °C | 100 | <1.0E-8 |  |
| 4-100-III | 4 °C | 100 | <1.0E-8 |  |
| 4-100-IV | 4 °C | 100 | <1.0E-8 |  |
| 4-10-I | 4 °C | 10 | <1.0E-8 |  |
| 4-10-II | 4 °C | 10 | <1.0E-8 |  |
| 4-10-III | 4 °C | 10 | <1.0E-8 |  |
| 4-10-IV | 4 °C | 10 | <1.0E-8 |  |
| 4-10-V | 4 °C | 10 | <1.0E-8 |  |
| 4-10-VI | 4 °C | 10 | <1.0E-8 |  |
| 4-10-VII | 4 °C | 10 | <1.0E-8 |  |
| 4-10-VIII | 4 °C | 10 | <1.0E-8 |  |
| 10-C-I | 10 °C | not infected | 5.81E-01 |  |
| 10-100-I | 10 °C | 100 | <1.0E-8 |  |
| 10-100-II | 10 °C | 100 | 3.6192E-05 |  |
| 10-100-III | 10 °C | 100 | 1.63E-04 |  |
| 10-10-I | 10 °C | 10 | 2.36E-04 |  |
| 10-10-II | 10 °C | 10 | 4.77E-04 |  |
| 10-10-III | 10 °C | 10 | 3.61E-04 |  |
| 20-100-I | 20 °C | 100 | 5.78E-04 |  |
| 20-10-I | 20 °C | 10 | 2.80E-04 |  |
| 20-10-II | 20 °C | 10 | 2.18E-04 |  |
|  |  |  |  |  |
| ^a^ Efficiency of plaquing (ratio of plaques formed on isolated selected of the long-term temperature experiment over plaques formed on sensitive *L. monocytogenes* EGDe) | | | | |
