# Supplementary material for: Influence of Environmental Factors on Phage–Bacteria Interaction and on the Efficacy and Infectivity of Phage P100
Source: Front Microbiol. 2016 Jul 28;7:1152. doi: 10.3389/fmicb.2016.01152 (PMC4964841; doi:10.3389/fmicb.2016.01152)
Supplement: Supplementary file 2 [file Data_Sheet_1.DOCX]

Supplementary Material

**Influence of environmental factors on phage-bacteria interaction and on the efficacy and infectivity of phage P100**

Susanne Fister^1^, Christian Robben^1^, Anna Kristina Witte^1^_,_ Dagmar Schoder^1,2^, Martin Wagner^2^, Peter Rossmanith^1,2*^

*** Correspondence:** Peter Rossmanith
University of Veterinary Medicine Vienna

Department for Farm Animals and Public Veterinary Health

Christian Doppler Laboratory for Monitoring of Microbial Contaminants

[peter.rossmanith@vetmeduni.ac.at](mailto:peter.rossmanith@vetmeduni.ac.at)

# Supplementary Figures and Tables

## Supplementary Figures


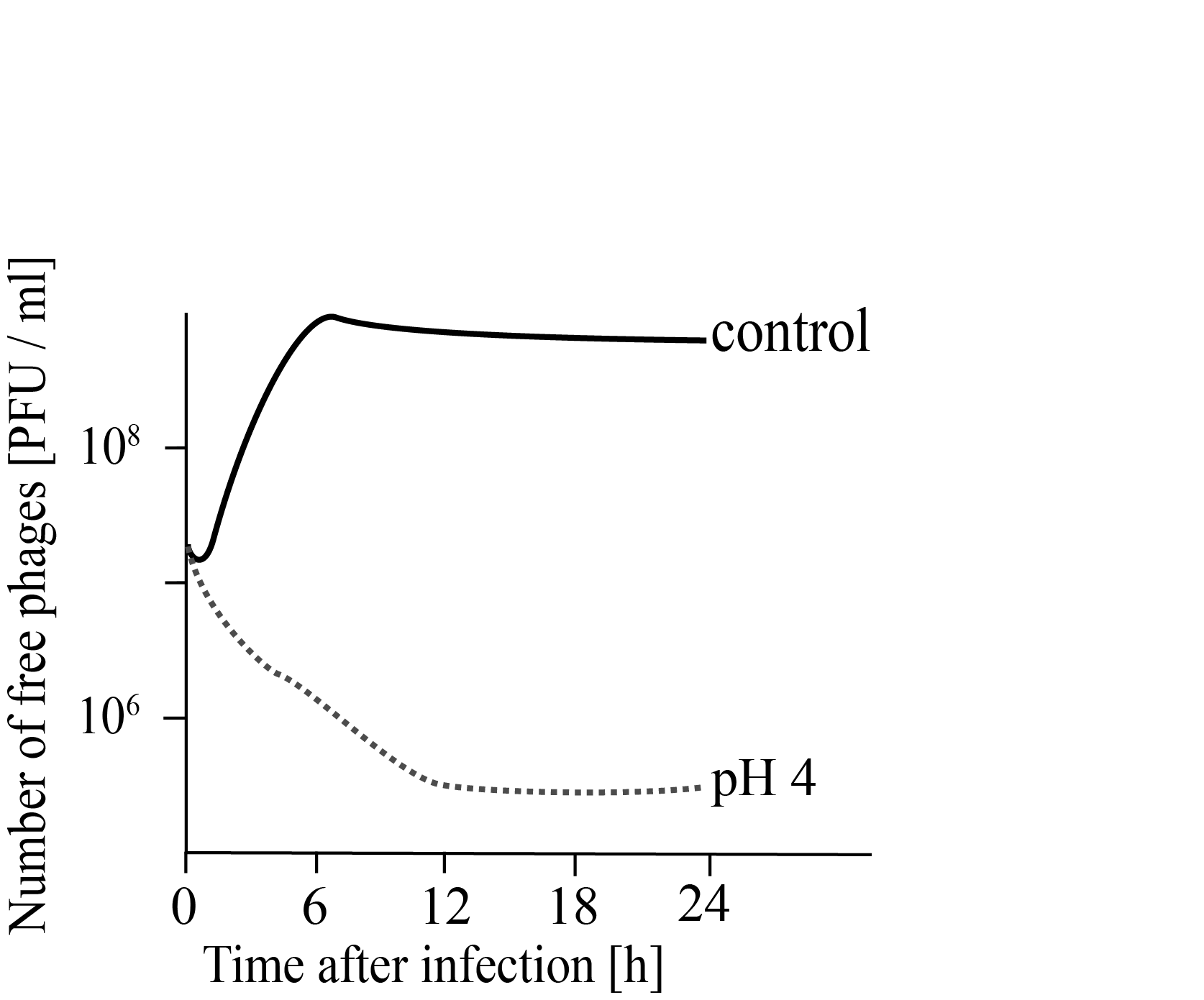


**Supplementary Figure 1**: Adsorption tests performed over 24 hours in TSB medium and TSB medium adjusted to pH 4. Graphs show the number of free (unattached extracellular) phages. The experiment was carried out twice and in duplicate.


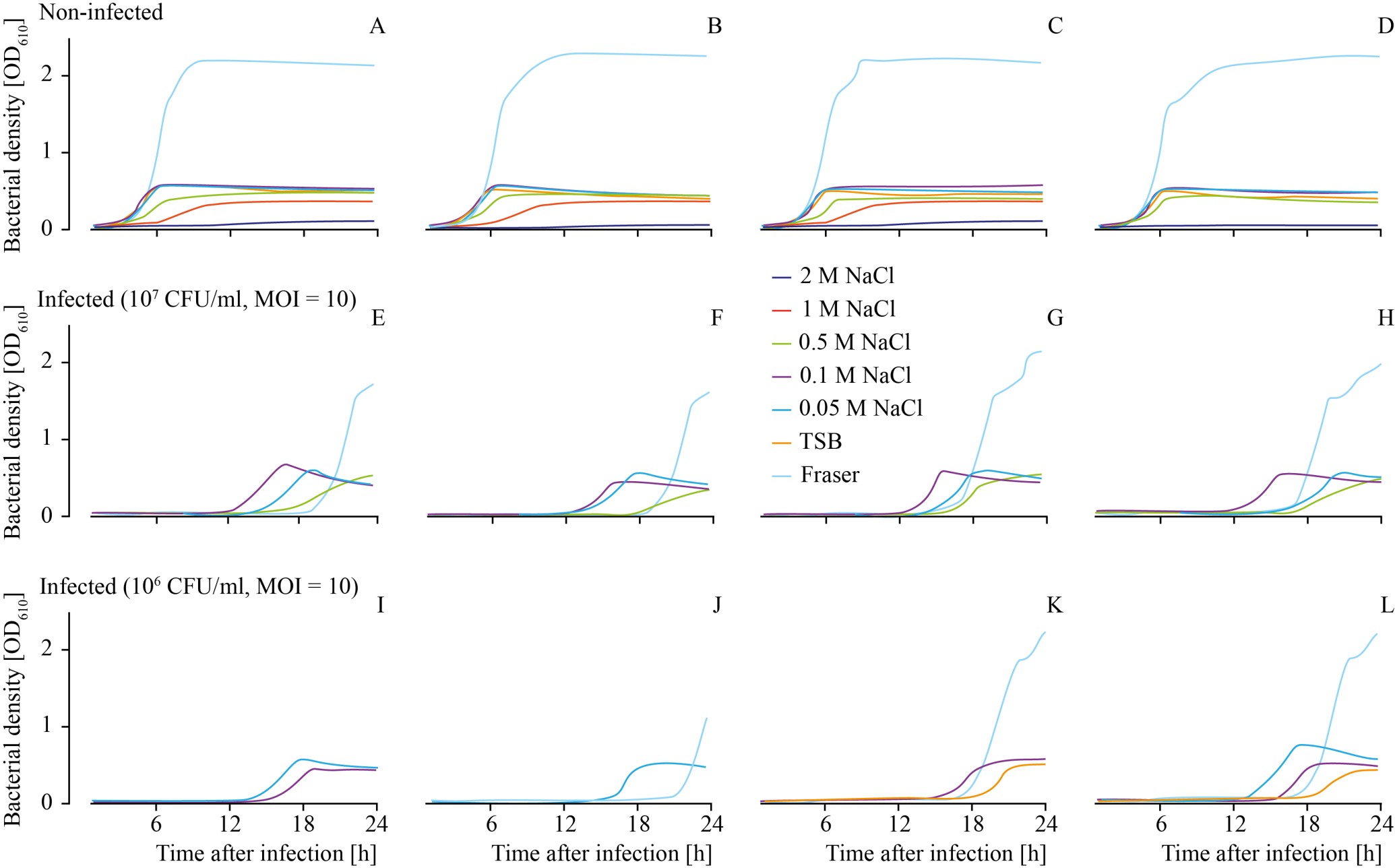
**Supplementary Figure 2.** Growth curves of uninfected (A-D) and infected (E -L; MOI = 10) *L. monocytogenes* in TSB, Fraser and NaCl containing TSB media. Bacteria concentrations at the beginning of the infections were 10^7^ CFU/ml (A -H) and 10^6^ CFU/ml (I-L). The high OD values of the F*raser* are caused not only by microbial growth. The presence of *Listeria spp.* additionally causes a colour change of the media that result in a higher optical density. All NaCl concentrations (0-2 M) and *Fraser* medium were tested in all 12 experiments. For the sake of clarity only the curves of growing bacteria were demonstrated while the others on base line level were not depicted.

**
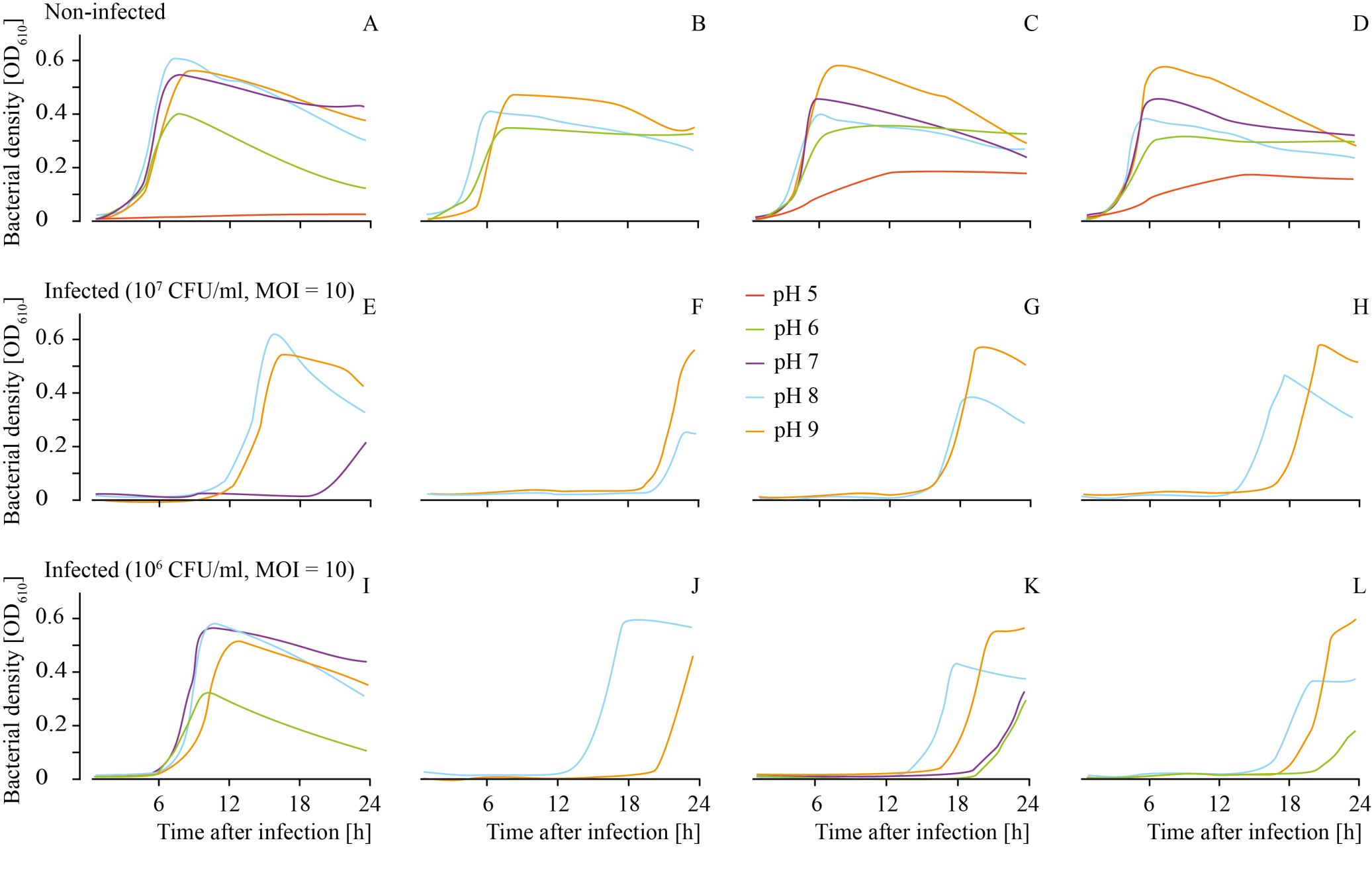
**

**Supplementary Figure 3.** Growth of uninfected (A-D) and infected (E-L; MOI = 10) *L. monocytogenes* in TSB adjusted to different pH values. The bacteria concentrations at the beginning of the infections were 10^7^ CFU/ml (A-H) and 10^6^ CFU/ml (I-L). All pH conditions were tested in all 12 experiments. For the sake of clarity only the curves of growing bacteria were demonstrated while the others on base line level were not depicted.


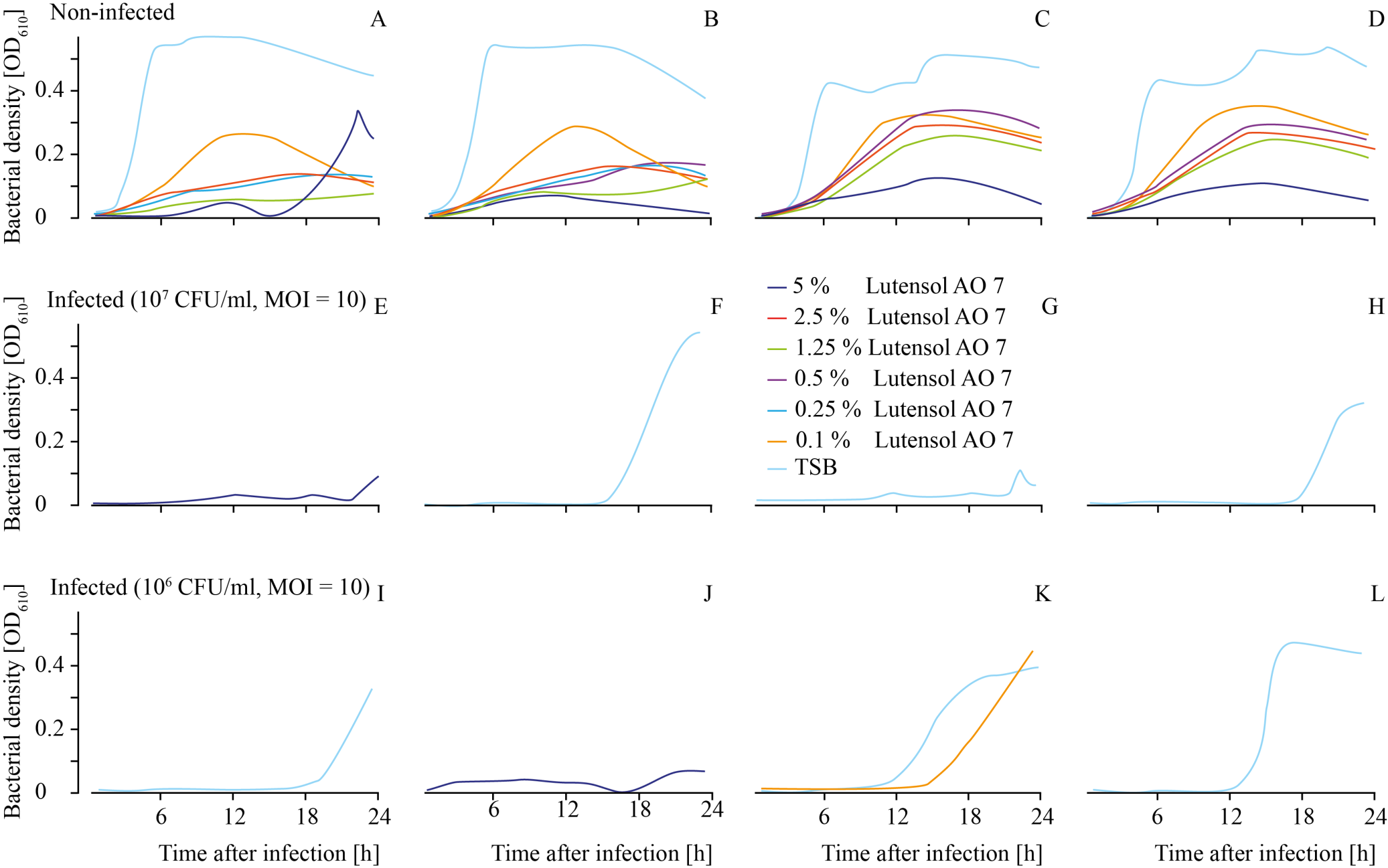


**Supplementary Figure 4.** Growth of uninfected (A-D) and infected (E-L; MOI = 10) *L. monocytogenes* in TSB adjusted to different pH values. The bacteria concentrations at the beginning of the infections were 10^7^ CFU/ml (A-H) and 10^6^ CFU/ml (I-L). All concentrations (0-5 %) were tested in all 12 experiments. For the sake of clarity only the curves of growing bacteria were demonstrated while the others on base line level were not depicted.

**Supplemantary Table 1:**

| Table S1: Isolates selected from long-term infection experiments | | | |  |
| --- | --- | --- | --- | --- |
|  |  |  |  |  |
| Isolate ID | Temperature | MOI | EOP^a^ |  |
|  |  |  |  |  |
|  |  |  |  |  |
| 4-C-I | 4 °C | not infected | 7.15E-01 |  |
| 4-100-I | 4 °C | 100 | <1.0E-8 |  |
| 4-100-II | 4 °C | 100 | <1.0E-8 |  |
| 4-100-III | 4 °C | 100 | <1.0E-8 |  |
| 4-100-IV | 4 °C | 100 | <1.0E-8 |  |
| 4-10-I | 4 °C | 10 | <1.0E-8 |  |
| 4-10-II | 4 °C | 10 | <1.0E-8 |  |
| 4-10-III | 4 °C | 10 | <1.0E-8 |  |
| 4-10-IV | 4 °C | 10 | <1.0E-8 |  |
| 4-10-V | 4 °C | 10 | <1.0E-8 |  |
| 4-10-VI | 4 °C | 10 | <1.0E-8 |  |
| 4-10-VII | 4 °C | 10 | <1.0E-8 |  |
| 4-10-VIII | 4 °C | 10 | <1.0E-8 |  |
| 10-C-I | 10 °C | not infected | 5.81E-01 |  |
| 10-100-I | 10 °C | 100 | <1.0E-8 |  |
| 10-100-II | 10 °C | 100 | 3.6192E-05 |  |
| 10-100-III | 10 °C | 100 | 1.63E-04 |  |
| 10-10-I | 10 °C | 10 | 2.36E-04 |  |
| 10-10-II | 10 °C | 10 | 4.77E-04 |  |
| 10-10-III | 10 °C | 10 | 3.61E-04 |  |
| 20-100-I | 20 °C | 100 | 5.78E-04 |  |
| 20-10-I | 20 °C | 10 | 2.80E-04 |  |
| 20-10-II | 20 °C | 10 | 2.18E-04 |  |
|  |  |  |  |  |
| ^a^ Efficiency of plaquing (ratio of plaques formed on isolated selected of the long-term temperature experiment over plaques formed on sensitive *L. monocytogenes* EGDe) | | | | |
